# Four-Objective Optimization for an Irreversible Porous Medium Cycle with Linear Variation in Working Fluid’s Specific Heat

**DOI:** 10.3390/e24081074

**Published:** 2022-08-03

**Authors:** Pengchao Zang, Lingen Chen, Yanlin Ge, Shuangshuang Shi, Huijun Feng

**Affiliations:** 1 Institute of Thermal Science and Power Engineering, Wuhan Institute of Technology, Wuhan 430205, China; zangpengchaoy@163.com (P.Z.); geyali9@hotmail.com (Y.G.); shishuangshuang20@163.com (S.S.); huijunfeng@139.com (H.F.); 2 Hubei Provincial Engineering Technology Research Center of Green Chemical Equipment, Wuhan 430205, China; 3 School of Mechanical & Electrical Engineering, Wuhan Institute of Technology, Wuhan 430205, China

**Keywords:** irreversible porous medium cycle, linear variable specific, power density, ecological function, multi-objective optimization, finite time thermodynamics

## Abstract

Considering that the specific heat of the working fluid varies linearly with its temperature, this paper applies finite time thermodynamic theory and NSGA-II to conduct thermodynamic analysis and multi-objective optimization for irreversible porous medium cycle. The effects of working fluid’s variable-specific heat characteristics, heat transfer, friction and internal irreversibility losses on cycle power density and ecological function characteristics are analyzed. The relationship between power density and ecological function versus compression ratio or thermal efficiency are obtained. When operating in the circumstances of maximum power density, the thermal efficiency of the porous medium cycle engine is higher and its size is less than when operating in the circumstances of maximum power output, and it is also more efficient when operating in the circumstances of maximum ecological function. The four objectives of dimensionless power density, dimensionless power output, thermal efficiency and dimensionless ecological function are optimized simultaneously, and the Pareto front with a set of solutions is obtained. The best results are obtained in two-objective optimization, targeting power output and thermal efficiency, which indicates that the optimal results of the multi-objective are better than that of one-objective.

## 1. Introduction

Finite time thermodynamics (FTT) [1,2,3,4,5,6,7,8,9,10,11] has been made significant progress in the research of thermal cycles and processes, including optimal configurations [12,13,14,15,16,17,18,19,20,21] and optimal performances [22,23,24,25,26,27,28,29,30,31,32]. The FTT studies of internal combustion engine cycles mostly focus on the following factors [33]: the effects of different loss models such as heat transfer loss (HTL) [34], friction loss (FL) [35] and internal irreversibility loss (IIL) [36] on the performances of cycles; the effects of power output (P) and thermal efficiency (η) [37], efficient power (Ep) [38], ecological function (E) [39], power density ($P_{d}$) [40] and other objective extreme values on the optimal performances of cycles; the effects of different working fluid (WF)-specific heat (SH) models on the performance of cycles, such as the constant SH of WF [41], the linear variable SH of WF [42] and the nonlinear variable SH of WF [43]; and the influence of WF quantum characteristics [44] and performance characteristics of universal cycle [45].

Many scholars have studied the P, η and Ep objective functions of the heat engine cycles. Diskin and Tartakovsky [46] combined electrochemical and Otto cycles, and studied the η characteristic relationship in the circumstances of maximum P. Wang et al. [47] investigated the P and η of Lenoir cycle. Bellos et al. [48] derived the η of a solar-fed organic Rankine cycle with reheating, which is more efficient than the conventional organic Rankine cycle. Gonca and Hocaoglu [49] investigated the Ep, Ep density and effective η of a Diesel–Miller cycle, considering the influences of compression ratio, pressure ratio and stroke ratio under the condition of variable SH of WF. Gonca and Sahin [50,51] combined the Miller cycle and the Takemura cycle, and derived the P, η, Ep, effective $P_{d}$, exergy destruction, exergy efficiency and ecological coefficient of the Miller–Takemura cycle.

Angulo-Brown et al. [52] first put forward the E as optimization objective (OO) in 1991 for heat engines. Yan [53] corrected E. Chen et al. [54] provided a unified definition of E for heat engines, refrigerators and heat pumps. Gonca and Genc [55] investigated the E, $P_{d}$, power generation and density of power generation of a gas–mercury–steam system. Jin et al. [56] optimized E performance of an irreversible recompression S-CO_2_ cycle and analyzed the influence of the mass flow rate, pressure ratio and diversion coefficient on E performance. Some researchers studied E performances for Brayton [38], diesel [57], Atkinson [58] and dual [59] as well as other cycles.

Sahin et al. [60] proposed the $P_{d}$ as OO for the first time and introduced it into the performance optimization of the reversible Joule-Brayton cycle. The numerical results show that the design parameters in the circumstances of maximum $P_{d}$ will result in smaller dimensions, higher η compared to maximum P circumstances. Al-Sarkhi et al. [61] investigated the $P_{d}$ characteristics of a Miller cycle when any loss does not need to be considered. With the $P_{d}$ as the OO, Gonca and Genc [62] optimized the double-reheat Rankine cycle which was based on a mercury turbine system. Gonca et al. [63] investigated the influence of the parameters, such as cycle intake temperature, intake pressure, pressure ratio and compression ratio, on the P, $P_{d}$ and exergy efficiency of a Dual-Diesel cycle. Gonca and Sahin [64] studied cycle P, $P_{d}$, ecological coefficient and effective ecological $P_{d}$ performances of a modified Dual cycle. Subsequently, the OO of $P_{d}$ [65,66,67] has been utilized in the performance research and optimizations of heat engines.

With the increase in OOs, there are contradictions among different OOs. To select the optimal result under the coexistence of multiple OOs, many scholars have carried out multi-objective optimization (MOO) [68,69,70,71,72,73,74,75,76,77] by NSGA-II [78]. Li et al. [68] established a regenerative Brayton cycle model and carried out MOO on the P, η and dimensionless thermal economic performance. Chen et al. [69] conducted MOO research on an irreversible modified closed Brayton cycle with four OOs of P, η, $P_{d}$ and E. Fergani et al. [70] performed MOO on the cyclohexane, toluene and benzene of an organic Rankine cycle using a multi-objective particle swarm optimizer. Teng et al. [71] performed MOO on the multiple systems under the conditions of different heat source temperatures of an organic Rankine cycle. Baghernejad et al. [72] took exergy efficiency, overall cost rate and exergy unit cost of generated electricity as OOs, and performed MOO on the combined Brayton and Rankine cycle. Xie et al. [73] performed MOO on the molar flow rate, reactor lengths and inlet temperatures of Braun-type exothermic reactor for ammonia synthesis. Shi et al. [74] and Ge et al. [75] used P, η, $P_{d}$ and E as OOs and performed MOO for the diesel [74], dual [75] and MHD [76] cycles.

Ferrenberg [79] first proposed a porous medium (PM) engine in 1990 and presented it as a regenerative engine. PM engine is a new type of engine based on PM combustion technology. Xie [80] introduced the super-adiabatic combustion technology in PM into the engine field and studied the characteristics of super-adiabatic combustion under reciprocating flow in PM. Waclas [81] divided the process of injecting high-pressure fuel into the PM body into four parts and proposed the idea of developing a low-emission engine. Durst and Weclas [82] modified a single-cylinder air-cooled diesel engine and proposed a design scheme for a PM engine. Generally, there are two working modes: one is the periodic contact between the PM and the cylinder, and the other is the permanent contact between the PM and the cylinder. PM engine has a larger internal surface area than other engines and are more capable of absorbing and storing heat. Compared with traditional gasoline or diesel engines, PM engines had higher η, lower emissions and higher P. Liu et al. [83] established the PM engine model with classical thermodynamic theory, and calculated the influence of compression ratio, pre-expansion ratio, pre-pressure ratio on the η and work output of the PM engine. Zhao et al. [84] investigated the effects of initial temperature, structure and injection duration on engine compression ignition in a methane-powered PM engine.

As one of the thermodynamic cycles, the PM cycle has constant volume processes in both endothermic and exothermic processes, similar to the Otto cycle. Liu et al. [85] first applied FTT theory to investigate P and η of an endoreversible PM cycle. Ge et al. [86] studied the P and η of an irreversible PM cycle. The PM cycle can be changed to the Otto cycle when the pre-expansion ratio is 1. Zang et al. [87] studied the $P_{d}$ performance and performed MOO of the P, η, $P_{d}$ and E of an irreversible PM cycle. 

The previous research of PM cycles assumed that the SH of the WF remained constant during the cycle, but in the actual cycle, the SH of the WF is constantly changing during the functioning of the heat engine. In this paper, based on Ref. [86], an irreversible PM cycle model will be established based on the linear change in SH of the working fluid with its temperature [88], and the FTT theory will be applied to further study the performance of $P_{d}$ and E. The η, $\bar{P}$, ${\bar{P}}_{d}$ and $\bar{E}$ of the irreversible PM cycle will be optimized by MOO, and the optimal result with the smallest deviation index (DI) will be obtained.

## 2. Model of an Irreversible PM Cycle

**Figure 1:**
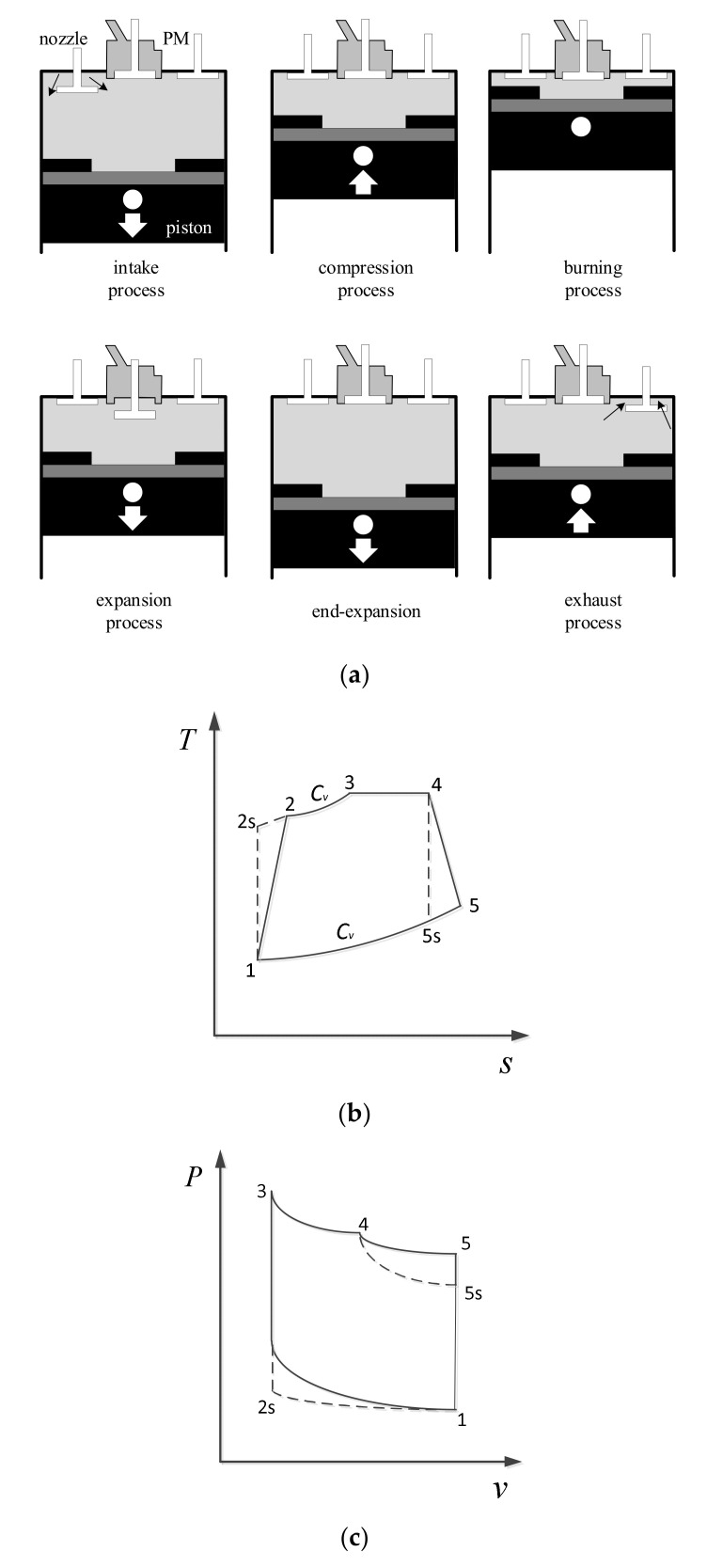
Model of PM cycle. (**a**) Working process of the PM engine. (**b**) T−s graphic. (**c**) P−v graphic.

The working process of the PM engine is shown in Figure 1a, and the PM combustion chamber is installed on the top of the cylinder. Fresh air enters the cylinder, at this time the PM chamber is isolated from the cylinder, and the PM chamber is fuel vapor. At the end of the intake process, the starter continues to drive the crankshaft to rotate, and the piston moves from bottom to top. At the same time, the PM chamber is closed, and the gas sucked into the cylinder by the intake stroke is enclosed in a closed space. The gas in the cylinder is compressed and the temperature and pressure are getting higher and higher At the end of the compression process, the valve of the PM chamber is opened, and the compressed air enters the PM chamber for instant recuperation, and the recuperation process is approximately a constant volume process. Air and fuel vapor are rapidly mixed in the PM chamber and self-ignited. The heat released during the combustion process is partly stored in the PM chamber and partly driven by the piston to do work, and the combustion process is approximately an isothermal endothermic process. At the end of the adiabatic expansion stroke, the PM chamber valve is closed. After the constant volume exhaust stroke, the intake stroke of a new cycle begins.

An irreversible PM cycle shown in Figure 1b,c: 1−2s is a reversible adiabatic compression process, 1−2 is an irreversible adiabatic compression process; 2−3 is a constant volume heat recovery process; 3−4 is an isothermal endothermic process; 4−5s is an reversible process of adiabatic expansion, 4−5 is an irreversible process of adiabatic expansion; and 5−1 is constant volume exothermic process.

In the actual cycle, the SH of the WF is constantly changing during the functioning of the heat engine. According to Ref. [88], when the working temperature of the heat engine is between 300K−2200K, the SH of the WF changes linearly with its temperature, and the constant volume SH of the WF is

(1) $$C_{v}=b_{v}+KT$$

where *b_v_* and *K* are constants.

The cycle temperature ratio (τ), pre-expansion ratio (ρ) and compression ratio (γ) are defined as

(2) $$\tau =T_{3}/T_{1}$$

(3) $$\rho =V_{4}/V_{3}$$

(4) $$\gamma =V_{1}/V_{2}$$

For processes 1−2 and 4−5, the IIL due to friction, turbulence and viscous stress of the cycle is represented by the compression and expansion efficiency:

(5) $$\eta_{c}=\left(T_{2S}-T_{1}\right)/\left(T_{2}-T_{1}\right)$$

(6) $$\eta_{e}=\left(T_{5}-T_{4}\right)/\left(T_{5S}-T_{4}\right)$$

Because the WF’s SH fluctuates with temperature, according to Ref. [88], it is assumed that the process can be decomposed into an infinite number of infinitesimal processes. For each infinitesimal process, it can be approximated that the SH is constant, adding all the infinitesimal processes together constitutes the entire adiabatic process, and any reversible adiabatic process between states i and j may be considered a reversible adiabatic process with infinitely small adiabatic exponent k as a constant. When the temperature and specific volume of the WF change by dT and dV, the following formula can be obtained

(7) $$TV^{k-1}=(T+dT){\left(V+dV\right)}^{k-1}$$

According to Equation (7), one has

(8) $$K(T_{j}-T_{i})+b_{v}\ln(T_{j}/T_{i})=-R\ln(V_{j}/V_{i})$$

According to the processes 1→2s and 4→5s, one has

(9) $$K(T_{2s}-T_{1})+b_{v}\ln(T_{2s}/T_{1})=R\ln\gamma$$

(10) $$K(T_{5s}-T_{4})+b_{v}\ln(T_{5s}/T_{4})=-R\ln(\gamma /\rho )$$

The heat absorption rate of WF is

(11) $${\dot{Q}}_{in}=M(\int_{T_{2}}^{T_{3}}C_{v}dT+RT_{3}\ln\rho )=M[b_{v}(T_{3}-T_{2})+0.5K(T_{3}^{2}-T_{2}^{2})+RT_{3}\ln\rho ]$$

The heat release rate of WF is

(12) $${\dot{Q}}_{out}=M\int_{T_{1}}^{T_{5}}C_{v}dT=M\int_{T_{1}}^{T_{5}}\left(b_{v}+KT)dT=M[b_{v}(T_{5}-T_{1})+0.5K(T_{5}^{2}-T_{1}^{2})\right]$$

where M is the mass flow rate.

In an actual PM cycle, there is HTL between the WF and the cylinder. According to Ref. [13], the HTL rate is defined as

(13) $${\dot{Q}}_{leak}=A-{\dot{Q}}_{in}=(B/2)\left(T_{2}+T_{3}-2T_{0}\right)=\left(T_{2}+T_{3}-2T_{0}\right)B_{1}$$

where A represents the fuel exothermic rate, $T_{0}$ represents ambient temperature and $B=2B_{1}$ represents the HTL coefficient.

The FL needs to be considered in an actual PM cycle. According to Ref. [35], the FL is a linear function of speed. The power dissipated by FL is

(14) $$P_{\mu }=4\mu {\left(4Ln\right)}^{2}=64\mu {\left(Ln\right)}^{2}$$

where n represents the rotational speed and L represents the stroke length.

The cycle P and η are

(15) $$\begin{array}{c} P_{}=Q_{in}-Q_{out}-P_{\mu }\\ =M[b_{v}(T_{1}+T_{3}-T_{2}-T_{5})+0.5K(T_{1}^{2}+T_{3}^{2}-T_{2}^{2}-T_{5}^{2})+RT_{3}\ln\rho ]-64\mu {\left(Ln\right)}^{2} \end{array}$$

(16) $$\begin{array}{c} \eta =\frac{P}{Q_{in}+Q_{leak}}\\ =\frac{M[b_{v}(T_{1}+T_{3}-T_{2}-T_{5})+0.5K(T_{1}^{2}+T_{3}^{2}-T_{2}^{2}-T_{5}^{2})+RT_{3}\ln\rho ]-64\mu {\left(Ln\right)}^{2}}{M[b_{v}(T_{3}-T_{2})+0.5K(T_{3}^{2}-T_{2}^{2})+RT_{3}\ln\rho ]+MB[T_{2}+T_{3}-2T_{0}]} \end{array}$$

According to Ref. [89], the volume of total cycle, stroke and clearance are, respectively, as follows:

(17) $$v_{t}=v_{s}+v_{c}$$

(18) $$v_{s}=\pi d^{2}L/4$$

(19) $$v_{c}=\pi d^{2}L/[4(\gamma -1)]$$

According to Ref. [60], the $P_{d}$ is defined as

(20) $$P_{d}=P/v_{max}=P/v_{1}=4(\gamma -1)M[C_{v}(T_{3}+T_{1}-T_{2}-T_{5})+RT_{3}\ln\rho ]/(\pi d^{2}L\gamma )$$

The entropy generation rates due to FL, HTL, IIL and exhaust stroke are, respectively:

(21) $$\sigma_{q}=B_{1}(T_{2}+T_{3}-2T_{0})[1/T_{0}-2/(T_{2}+T_{3})]$$

(22) $$\sigma_{\mu }=\frac{P_{\mu }}{T_{0}}=\frac{64\mu {\left(Ln\right)}^{2}}{T_{0}}$$

(23) $$\sigma_{2S\to 2}=MC_{v}\ln\frac{T_{2}}{T_{2S}}=MC_{v}\ln\frac{T_{2}}{\eta_{c}(T_{2}-T_{1})+T_{1}}$$

(24) $$\sigma_{5S\to 5}=MC_{v}\ln\frac{T_{5}}{T_{5S}}=MC_{v}\ln\frac{\eta_{e}T_{5}}{T_{5}+(\eta_{e}-1)T_{4}}$$

(25) $$\sigma_{pq}=M\int_{T_{1}}^{T_{5}}C_{v}dT(\frac{1}{T_{0}}-\frac{1}{T})=M[\frac{C_{v}(T_{5}-T_{1})}{T_{0}}-C_{v}\ln\frac{T_{5}}{T_{1}}]$$

The total entropy generation rate is

(26) $$\begin{array}{c} \sigma =\sigma_{q}+\sigma_{\mu }+\sigma_{2S\to 2}+\sigma_{5S\to 5}+\sigma_{pq}\\ =[MB(T_{2}+T_{3}-2T_{0})+64\mu (Ln{)}^{2}]/T_{0}\\ +M[C_{v2S\to 2}\ln(T_{2}/T_{2S})+C_{v5S\to 5}\ln(T_{5}/T_{5S})]\\ +M\{[b_{v}(T_{5}-T_{1})/T_{0}]-b_{v}\ln(T_{5}/T_{1})+0.5K(T_{5}^{2}-T_{1}^{2})/(2T_{0})-K(T_{5}-T_{1})\} \end{array}$$

In Equation (26), the temperature in constant volume SH ($C_{v2S\to 2}$) is $T=\frac{T_{2}-T_{2S}}{\ln(T_{2}/T_{2S})}$, and the temperature in constant volume SH ($C_{v5S\to 5}$) is $T=\frac{T_{5}-T_{5S}}{\ln(T_{5}/T_{5S})}$.

The cycle E is

$$E=P-T_{0}\sigma$$

(27) $$\begin{array}{l} =M[b_{v}(T_{1}+T_{3}-T_{2}-T_{5})+0.5k(T_{1}^{2}+T_{3}^{2}-T_{2}^{2}-T_{5}^{2})+RT_{3}\ln\rho ]\\ -MB(T_{2}+T_{3}-2T_{0})(1-2T_{0}/(T2+T3))-128\mu (Ln{)}^{2}\\ -MT_{0}[C_{v2S\to 2}\ln(T_{2}/T_{2S})+C_{v5S\to 5}\ln(T_{5}/T_{5S})]-M[b_{v}(T_{5}\\ -T_{1})-b_{v}T_{0}\ln(T_{5}/T_{1})+0.5K(T_{5}^{2}-T_{1}^{2})-T_{0}K(T_{5}-T_{1})] \end{array}$$

In the actual cycle, the state 3 must be between states 2 and 4, so ρ should satisfy:

(28) $$1\leq \rho \leq V_{4}/V_{2}$$

According to Ref. [86], PM cycle converts to the Otto cycle when ρ=1, and the P, η, $P_{d}$, and E expressions of the Otto cycle can be derived from Equations (15), (16), (20) and (27).

The P, $P_{d}$ and E after dimensionless treatment are, respectively:

(29) $$\bar{P}=P/P_{\max}$$

(30) $${\bar{P}}_{d}=P_{d}/{\left(P_{d}\right)}_{\max}$$

(31) $$\bar{E}=E/E_{\max}$$

Given the γ, the initial temperature $T_{1}$, the ρ, the maximum cycle temperature $T_{4}$, the $\eta_{c}$ and $\eta_{e}$, the Equation (9) can be used to solve $T_{2S}$. Then solve $T_{2}$ from Equation (5), solve $T_{5S}$ from Equation (10), and finally solve $T_{5}$ from Equation (6). By substituting the solved $T_{2}$ and $T_{5}$ into Equations (15), (16), (20) and (27), you can obtain the corresponding P, η, $P_{d}$ and E.

## 3. Power Density and Ecological Functions Analyses and Optimizations

The parameters are determined according to Refs. [75,86]: ρ=1.2, τ=5.78~6.78,$b_{v}=19.868-23.868J/\mathrm{mol}.K$, $k_{1}=0.003844-0.009844J/\mathrm{mol}{.K}^{2}$, $T_{0}=300K$, $T_{1}=350K$, μ=1.2kg/s, $\dot{M}=1\mathrm{mol}/s$, B=2.2W/K, L=0.07m and $n=30s^{-1}$.

### 3.1. Power Density Analyses and Optimizations

**Figure 2:**
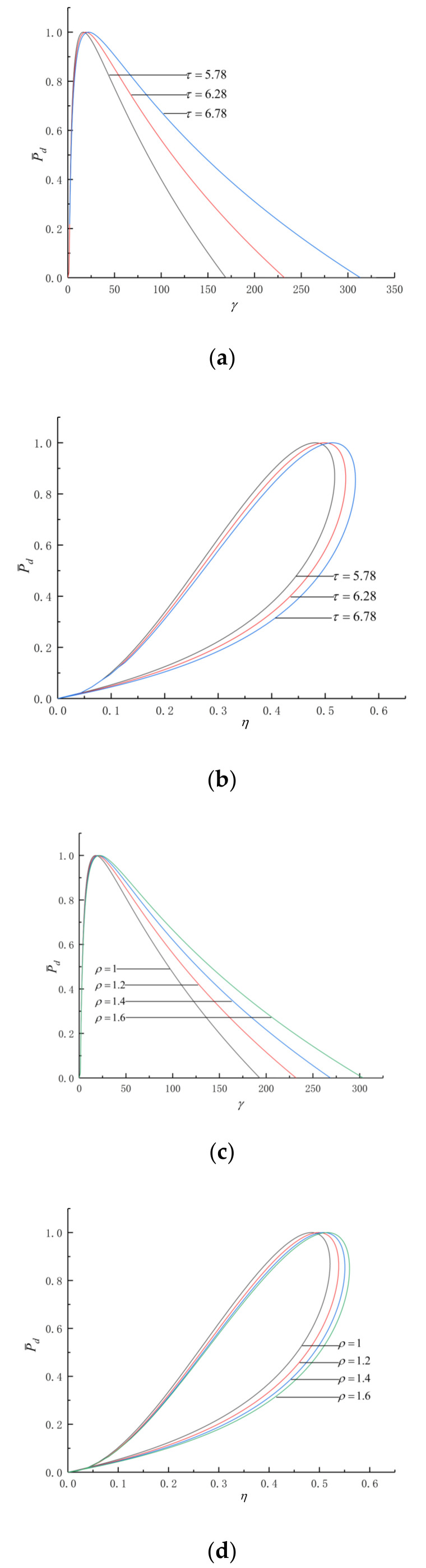
The effects of τ and ρ on ${\bar{P}}_{d}-\gamma$ and ${\bar{P}}_{d}-\eta$. (**a**) Effect of τ on ${\bar{P}}_{d}-\gamma$. (**b**) Effect of τ on ${\bar{P}}_{d}-\eta$. (**c**) Effect of ρ on ${\bar{P}}_{d}-\gamma$. (**d**) Effect of ρ on ${\bar{P}}_{d}-\eta$.

Figure 2 shows the effects of τ and ρ on the ${\bar{P}}_{d}$ and γ (${\bar{P}}_{d}-\gamma$) as well as the ${\bar{P}}_{d}$ and η (${\bar{P}}_{d}-\eta$) characteristics. The curve of ${\bar{P}}_{d}-\gamma$ is parabolic-like one, and the ${\left({\bar{P}}_{d}\right)}_{\max}$ corresponds to a optimal γ ($\gamma_{{\bar{P}}_{d}}$). The curve of ${\bar{P}}_{d}-\eta$ is loop-shaped one which starts from the origin and back to the origin, and there are operating points of ${\left({\bar{P}}_{d}\right)}_{\max}$ and maximum η ($\eta_{\max}$) in the cycle.

As seen in Figure 2a,b, as τ grows, both $\gamma_{{\bar{P}}_{d}}$ and $\eta_{{\bar{P}}_{d}}$ get larger. When τ grows from 5.78 to 6.78, $\gamma_{{\bar{P}}_{d}}$ grows from 16.5 to 22.3, $\eta_{{\bar{P}}_{d}}$ grows from 0.4809 to 0.5139 and $\eta_{{\bar{P}}_{d}}$ grows by about 6.86%. As seen in Figure 2c,d, as ρ grows, both $\gamma_{{\bar{P}}_{d}}$ and $\eta_{{\bar{P}}_{d}}$ get larger. When ρ grows from 1.2 to 1.6, $\gamma_{{\bar{P}}_{d}}$ grows from 19.3 to 21.9, $\eta_{{\bar{P}}_{d}}$ grows from 0.4986 to 0.5154 and $\eta_{{\bar{P}}_{d}}$ grows by about 3.37%. With the increase in the temperature ratio and pre-expansion ratio, the compression ratio and thermal efficiency in the circumstances of maximum dimensionless power density increase. In Figure 2, ρ=1 is the performance characteristics of the Otto cycle. Obviously, the PM cycle has a higher η than the Otto cycle. 

**Figure 3:**
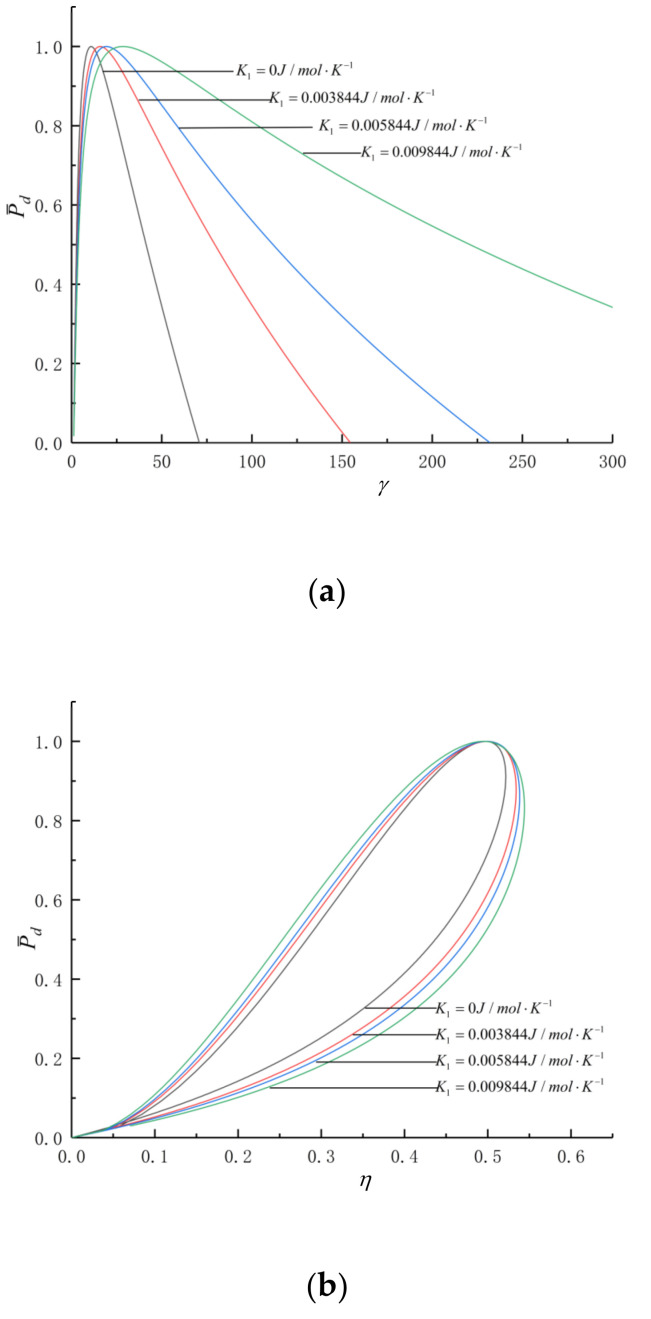

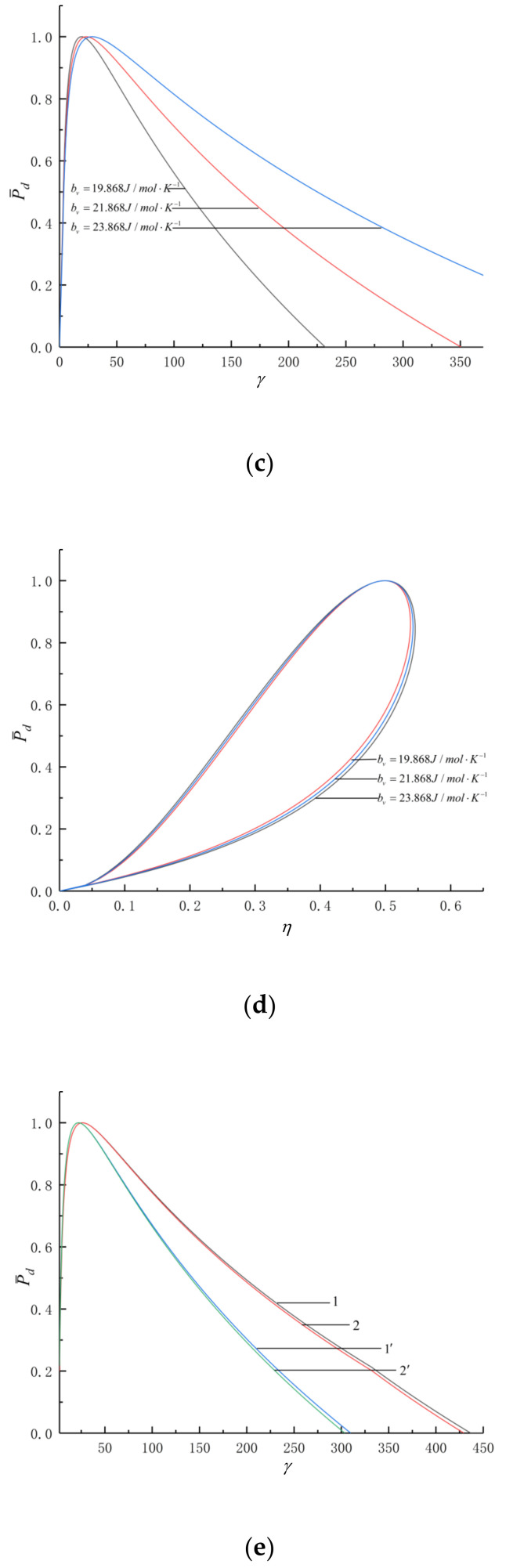

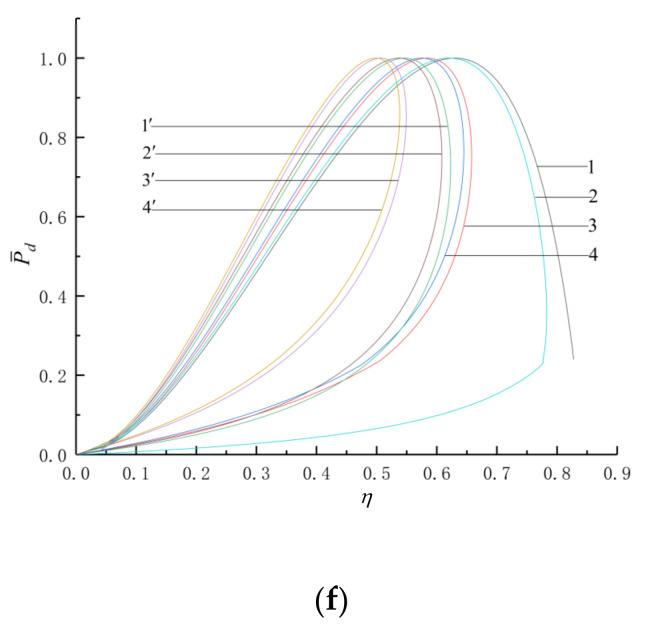
The effects of $k_{1}$ $b_{v}$ B μ $\eta_{c}$ and $\eta_{e}$ on ${\bar{P}}_{d}-\gamma$ and ${\bar{P}}_{d}-\eta$. (**a**) Effect of $k_{1}$ on ${\bar{P}}_{d}-\gamma$. (**b**) Effect of $k_{1}$ on ${\bar{P}}_{d}-\eta$. (**c**) Effect of $b_{v}$ on ${\bar{P}}_{d}-\gamma$. (**d**) Effect of $b_{v}$ on ${\bar{P}}_{d}-\eta$. (**e**) ${\bar{P}}_{d}-\gamma$. (**f**) ${\bar{P}}_{d}-\eta$.

Figure 3 shows the ${\bar{P}}_{d}-\gamma$ and ${\bar{P}}_{d}-\eta$ curves with varying losses and SH characteristics.

Figure 3a,b show the effects of $k_{1}$ on (${\bar{P}}_{d}-\gamma$) and (${\bar{P}}_{d}-\eta$) characteristics. The degree of variation in the SH of the WF with temperature is represented by $k_{1}$. The larger the $k_{1}$, the larger the variation range of the SH. As $k_{1}$ grows, $\gamma_{{\bar{P}}_{d}}$ grows and $\eta_{{\bar{P}}_{d}}$ declines. When $k_{1}=0$, the cycle WF is constant SH. When $k_{1}$ grows from $0.003844J/\mathrm{mol}{.K}^{2}$ to $0.009844J/\mathrm{mol}{.K}^{2}$, $\gamma_{{\bar{P}}_{d}}$ grows from 15.8 to 28.4, $\eta_{{\bar{P}}_{d}}$ declines from 0.4992 to 0.4949, a decline of 0.86%. Figure 3c,d show the effects of $b_{v}$ on ${\bar{P}}_{d}-\gamma$ and ${\bar{P}}_{d}-\eta$ characteristics. As $b_{v}$ grows, both $\gamma_{{\bar{P}}_{d}}$ and $\eta_{{\bar{P}}_{d}}$ will become larger. When $b_{v}$ grows from 19.868J/mol.K to 23.868J/mol.K, $\gamma_{{\bar{P}}_{d}}$ grows from 19.3 to 28.4, $\eta_{{\bar{P}}_{d}}$ grows from 0.4986 to 0.4993 and $\eta_{{\bar{P}}_{d}}$ grows by about 0.14%. As seen in Figure 3e,f, when only FL exists, comparing curves 1 and 2, as μ grows from 0kg/s to 1.2kg/s, $\gamma_{{\bar{P}}_{d}}$ is nearly unchanged, and $\eta_{{\bar{P}}_{d}}$ declines from 62.95% to 62.03%, a decline of 1.46%. When IIL exists only, comparing curves 1 and $1^{\prime }$, as $\eta_{c}$ and $\eta_{e}$ declines from 1 to 0.94, $\gamma_{{\bar{P}}_{d}}$ declines from 22.9 to 19.3, $\eta_{{\bar{P}}_{d}}$ declines from 62.95% to 54.65%, a decline of 13.19%. When only HTL exists, comparing curves 1 and 3, as B grows from 0W/K to 2.2W/K, $\eta_{{\bar{P}}_{d}}$ declines from 62.95% to 58.34%, a decline of 7.32%. When μ, $\eta_{c}$ and $\eta_{e}$ exist, comparing curves 1 and $2^{\prime }$, as μ grows from 0kg/s to 1.2kg/s, and the $\eta_{c}$ and $\eta_{e}$ decline from 1 to 0.94, $\gamma_{{\bar{P}}_{d}}$ declines from 22.9 to 19.3, $\eta_{{\bar{P}}_{d}}$ declines from 62.95% to 53.74%, a decline of 14.63%. When FL and HTL exist, comparing curves 1 and 4, as μ grows from 0kg/s to 1.2kg/s, and B grows from 0W/K to 2.2W/K, $\eta_{{\bar{P}}_{d}}$ declines from 62.95% to 57.49%, a decline of 8.67%. When IIL and HTL exist, comparing curves 1 and $3^{\prime }$, as $\eta_{c}$ and $\eta_{e}$ decline from 1 to 0.94, the B grows from 0W/K to 2.2W/K, $\gamma_{{\bar{P}}_{d}}$ declines from 22.9 to 19.3, $\eta_{{\bar{P}}_{d}}$ declines from 62.95% to 50.71%, a decline of 19.44%. When FL, HTL and IIL exist, comparing curves 1 and $4^{\prime }$, as μ grows from 0kg/s to 1.2kg/s, the B grows from 0W/K to 2.2W/K, and the $\eta_{c}$ and $\eta_{e}$ decline from 1 to 0.94, $\gamma_{{\bar{P}}_{d}}$ declines from 22.9 to 19.3, $\eta_{{\bar{P}}_{d}}$ declines from 62.95% to 49.86%, a decline of 20.79%. As the specific heat of the working fluid changes more violently with temperature and the three losses increase, the thermal efficiency in the circumstances of maximum dimensionless power density decreases.

**Figure 4:**
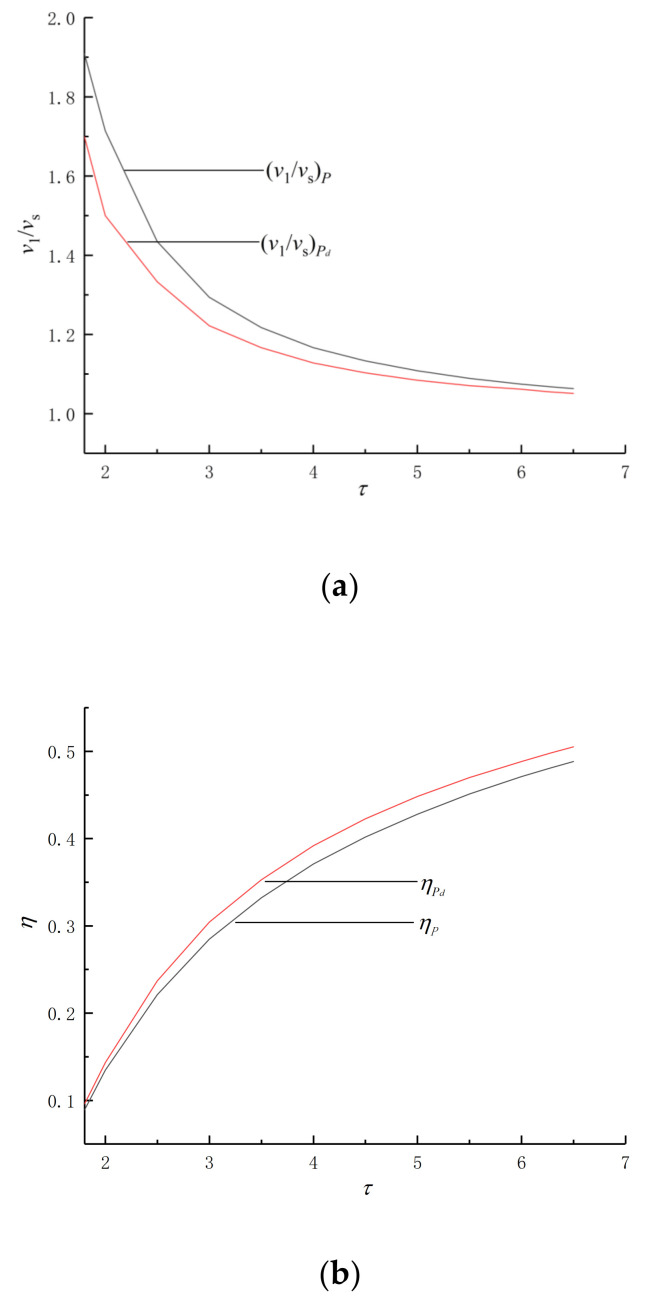

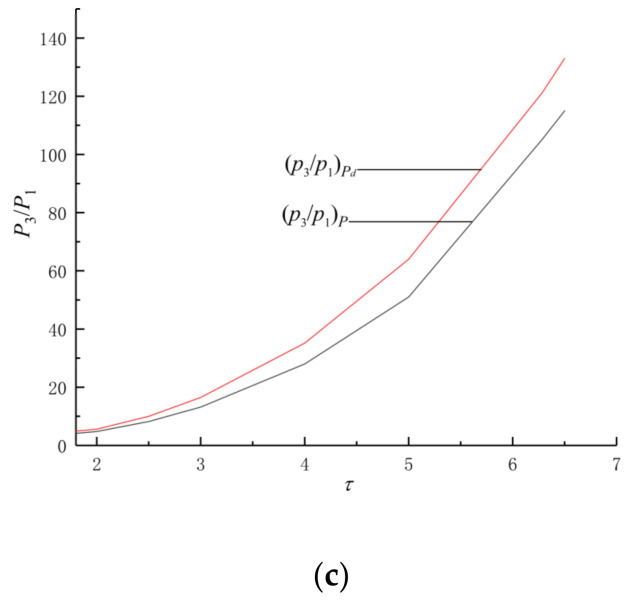
Various variations in $v_{1}/v_{s}$, η and $p_{3}/p_{1}$ with τ. (**a**) $v_{1}/v_{s}$ with τ. (**b**) η with τ. (**c**) $p_{3}/p_{1}$ with τ.

Figure 4 shows the variation in maximum-specific volume ratio ($v_{1}/v_{s}$), η and maximum pressure ratio ($p_{3}/p_{1}$) with τ in the circumstances of ${\bar{P}}_{\max}$ and ${\left({\bar{P}}_{d}\right)}_{\max}$. Figure 4a shows the $v_{1}/v_{s}$, where $v_{1}$ is the maximum-specific volume, $v_{s}$ is the stroke volume, and the larger the $v_{1}/v_{s}$, the larger the volume of the engine. Figure 4c shows the $p_{3}/p_{1}$, $p_{3}$ is the maximum pressure of the cycle, $p_{1}$ is the minimum pressure of the cycle, the larger the $p_{3}/p_{1}$, the higher the internal pressure of the engine, and the higher the requirements for engine materials. 

The $v_{1}/v_{s}$ corresponding to ${\bar{P}}_{\max}$ is always larger than $v_{1}/v_{s}$ corresponding to ${\left({\bar{P}}_{d}\right)}_{\max}$, the $p_{3}/p_{1}$ corresponding to ${\left({\bar{P}}_{d}\right)}_{\max}$ is always larger than the $p_{3}/p_{1}$ ratio corresponding to ${\bar{P}}_{\max}$ and $\eta_{{\bar{P}}_{d}}$ is always higher than $\eta_{\bar{P}}$. Compared with ${\bar{P}}_{\max}$, the cycle in the circumstances of ${\left({\bar{P}}_{d}\right)}_{\max}$ is smaller and more efficient.

### 3.2. Ecological Function Analyses and Optimizations

**Figure 5:**
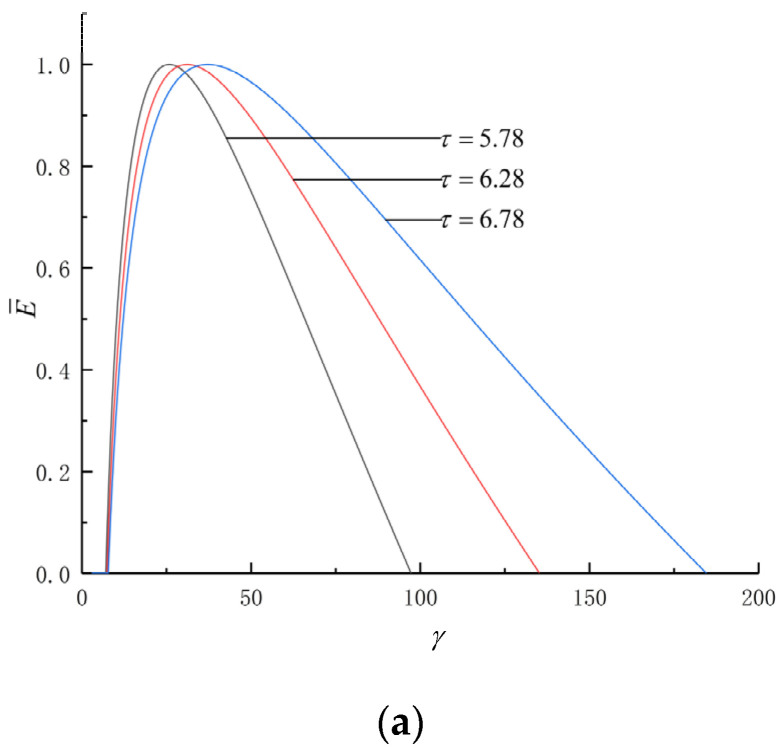

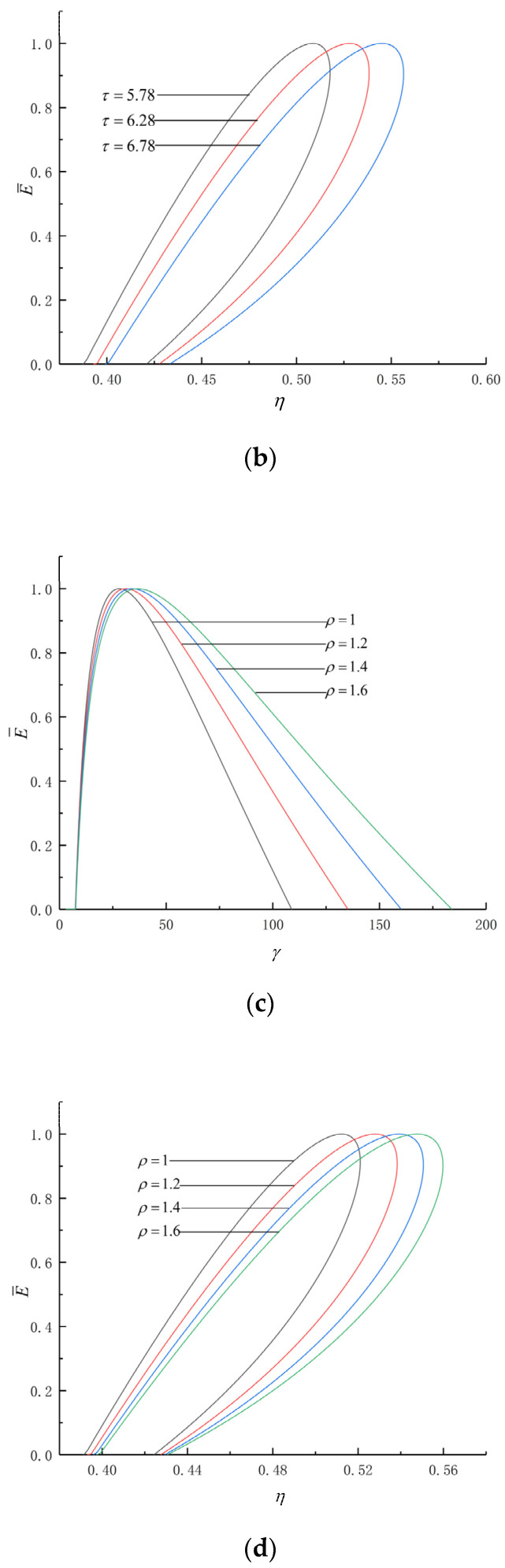
The effects of τ and ρ on $\bar{E}-\gamma$ and $\bar{E}-\eta$. (**a**) Effect of τ on $\bar{E}-\gamma$. (**b**) Effect of τ on $\bar{E}-\eta$. (**c**) Effect of ρ on $\bar{E}-\gamma$. (**d**) Effect of ρ on $\bar{E}-\eta$.

Figure 5 shows the effects of cycle parameters on the $\bar{E}$ and γ ($\bar{E}-\gamma$) as well as the $\bar{E}$ and η ($\bar{E}-\eta$) characteristics. It can be seen that the $\bar{E}-\gamma$ is parabolic-like one, and the maximum ecological function (${\bar{E}}_{\max}$) corresponds to a γ of $\gamma_{\bar{E}}$.The $\bar{E}-\eta$ is loop-shaped one, and there is an ${\bar{E}}_{\max}$ operating point and an $\eta_{\max}$ operating point in the cycle As seen in Figure 5a,b, as τ grows, both $\gamma_{\bar{E}}$ and $\eta_{\bar{E}}$ get larger. When τ grows from 5.78 to 6.78, $\gamma_{\bar{E}}$ grows from 25.8 to 37.1, $\eta_{\bar{E}}$ grows from 0.5086 to 0.5450 and $\eta_{\bar{E}}$ grows by about 7.16%. As seen in Figure 5c,d, as ρ grows, both $\gamma_{\bar{E}}$ and $\eta_{\bar{E}}$ get larger. When ρ grows from 1.2 to 1.6, $\gamma_{\bar{E}}$ grows from 33.5 to 43.6, $\eta_{\bar{E}}$ grows from 0.5303 to 0.5634 and $\eta_{\bar{E}}$ grows by about 3.37%. With the increase in the temperature ratio and pre-expansion ratio, the compression ratio and thermal efficiency in the circumstances of maximum dimensionless ecological function increase.

**Figure 6:**
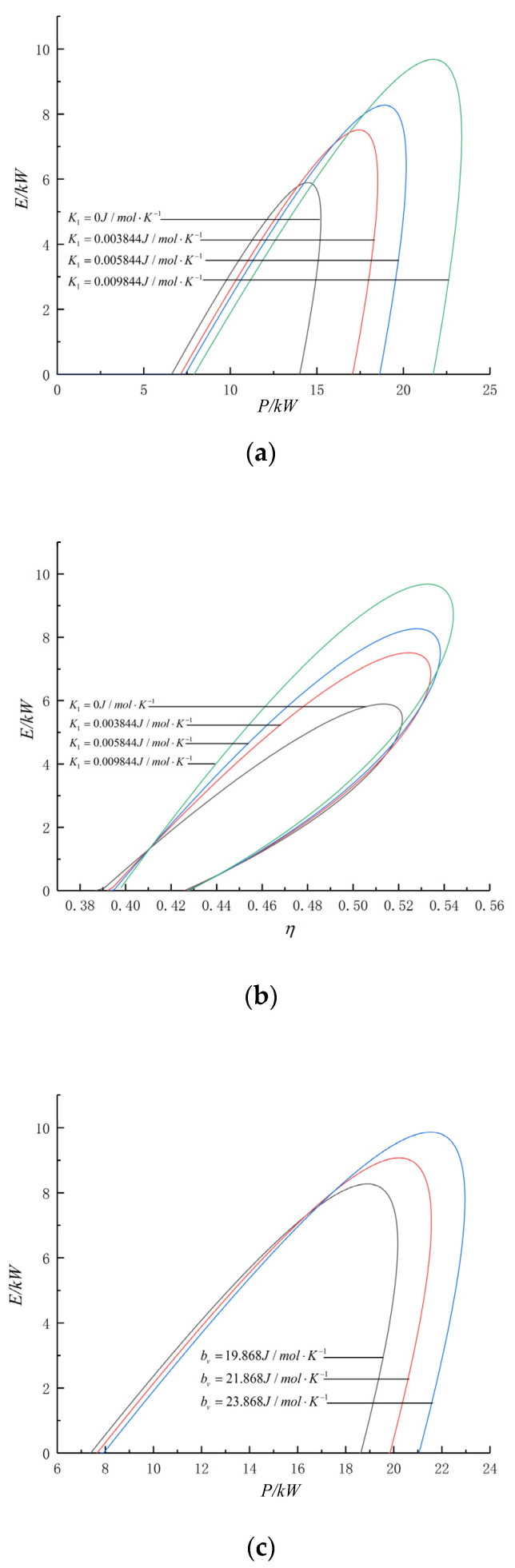

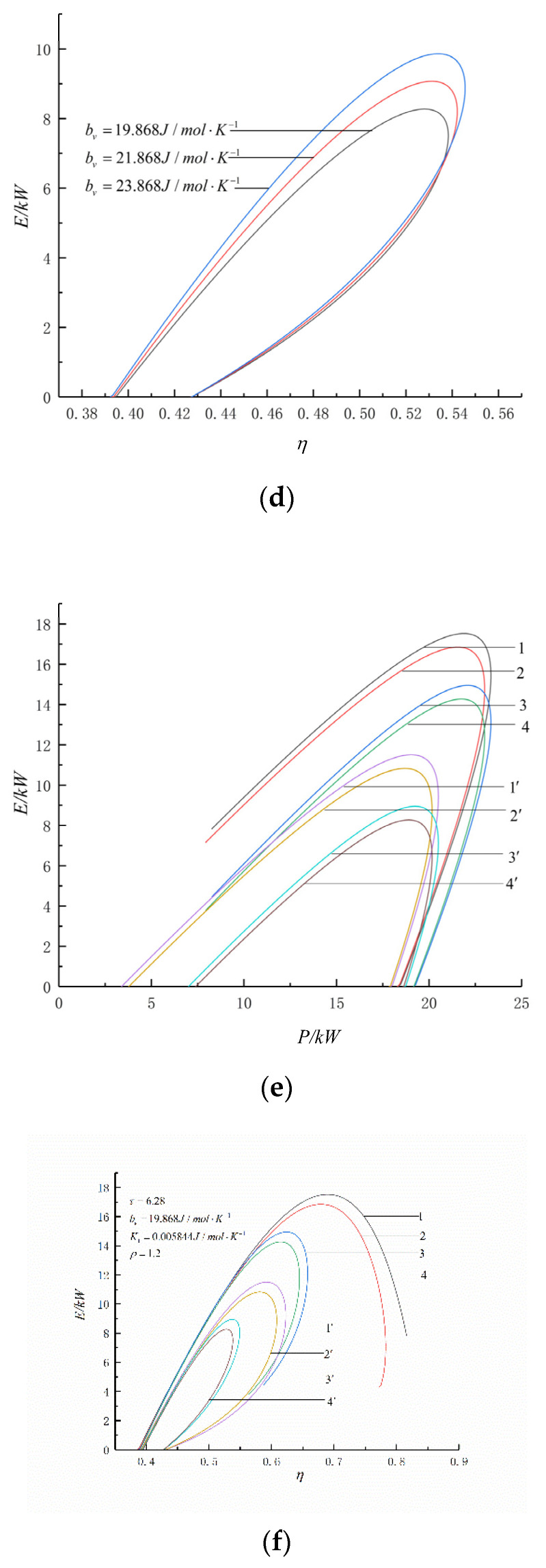
Effects of $k_{1}$, $b_{v}$, B, μ, $\eta_{c}$ $\eta_{e}$ on ${\bar{P}}_{d}-\gamma$ and ${\bar{P}}_{d}-\eta$. (**a**) Effect of $k_{1}$ on E-P. (**b**) Effect of $k_{1}$ on E-η. (**c**) Effect of $b_{v}$ on E-P. (**d**) Effect of $b_{v}$ on E-η. (**e**) E-P. (**f**) E-η.

Figure 6 shows the E−P and E−η curves with varying losses and SH characteristics. Figure 6a,c and e show that, except at the $P_{\max}$ point, corresponding to any E of the cycle, the P has two different values. The E of the cycle decreases with increasing μ, B, $\eta_{c}$ and $\eta_{e}$. Curve 1 in Figure 6f is reversible without any loss, and the curve is a parabolic-like one, whereas the others are loop-shaped. Each E value (except the maximum value point) corresponds to two η values. The heat engine should be run in the circumstances with a higher η during actual operation. Figure 6a–d show the effects of SH of WF characteristics on cycle performance. Among them, curve 1 is the E-P of the heat engine and the E-η under the conditions of constant SH of WF. Under certain conditions of ecological function, the PM heat engine should be run at a larger power output during actual operation. As the specific heat of the working fluid changes more violently with temperature and the three losses decrease, the ecological function, power output and thermal efficiency will all increase.

**Figure 7:**
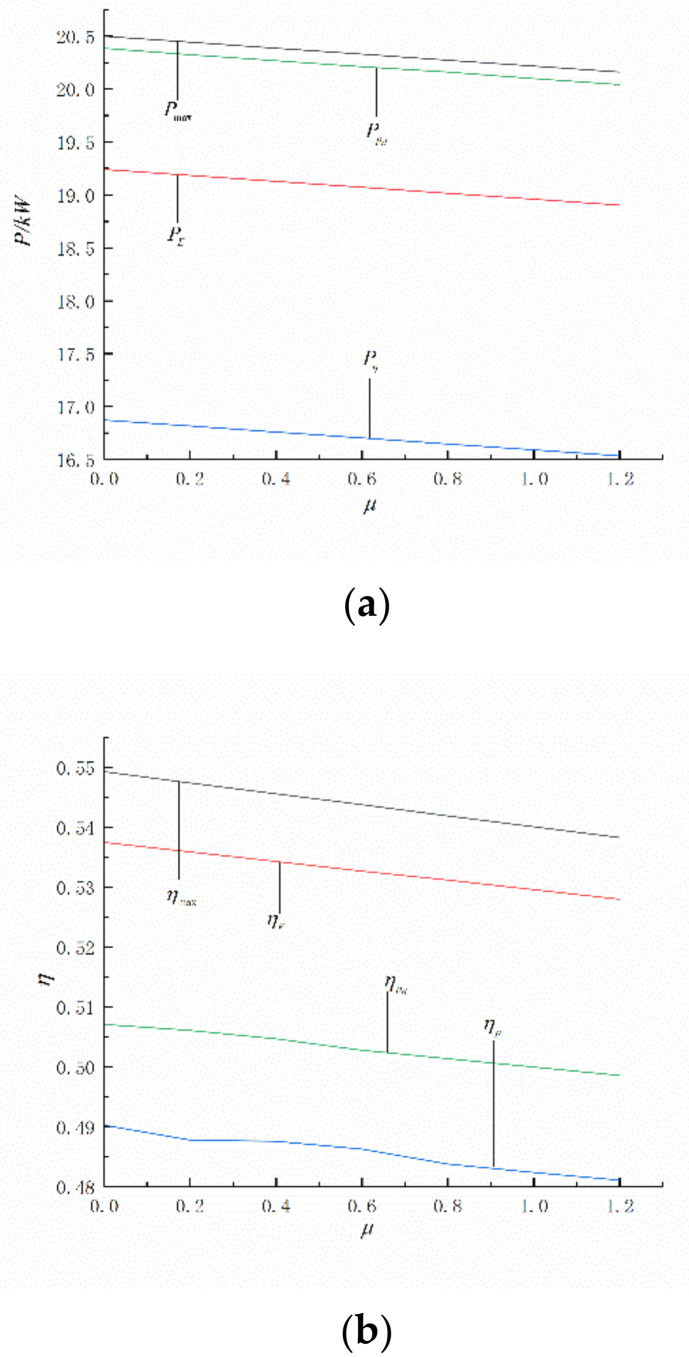
P and η in the circumstances of different objective functions. (**a**) P. (**b**) η.

Figure 7 shows the relationship between P and η characteristics under different OOs. Through numerical calculations, the $P_{\max}$, $\eta_{\max}$, P in the circumstances of $\eta_{\max}$ ($P_{\eta }$), P in the circumstances of ${\bar{E}}_{\max}$ ($P_{E}$), P in the circumstances of ${\left({\bar{P}}_{d}\right)}_{\max}$ ($P_{p_{d}}$), η in the circumstances of $P_{\max}$ ($\eta_{p}$), η in the circumstances of ${\left({\bar{P}}_{d}\right)}_{\max}$ ($\eta_{p_{d}}$), and η in the circumstances of $E_{\max}$ ($\eta_{E}$) can be obtained. Both P and η decline with the increases of μ, and $P_{\max}$>$P_{p_{d}}$>$P_{E}$>$P_{\eta }$, $\eta_{\max}$>$\eta_{E}$>$\eta_{p_{d}}$>$\eta_{p}$. Numerical calculations show that when the μ is 1.2kg/s, $P_{\max}$ is 20162 W, $P_{p_{d}}$ is 20049 W, $P_{E}$ is 18904 W, $P_{\eta }$ is 16725 W, $\eta_{\max}$ is 0.5383 W, $\eta_{p_{d}}$ is 0.4986 W, $\eta_{E}$ is 0.5280, and $\eta_{p}$ is 0.4811. Compared with $P_{\max}$, $P_{p_{d}}$ decreased by about 0.56%, $P_{E}$ decreased by about 6.23%, and $P_{\eta }$ decreased by about 17.05%. Compared with $\eta_{\max}$, $\eta_{p_{d}}$ decreased by about 7.38%, $\eta_{E}$ decreased by about 1.91%, $\eta_{p}$ decreased by about 10.63%. Compared with $E_{\max}$, $P_{p_{d}}$ decreased by about 5.71%, $\eta_{p_{d}}$ increased by about 5.57%. $P_{p_{d}}$ and $P_{E}$ are higher than $P_{\eta }$, $\eta_{E}$ and $\eta_{p_{d}}$ are higher than $\eta_{p}$**,** $P_{p_{d}}$ is higher than $P_{E}$ and $\eta_{E}$ is higher than $\eta_{p_{d}}$. The ecological function objective function reflects the compromise between power output and efficiency.

## 4. Multi-Objective Optimizations

**Figure 8:**
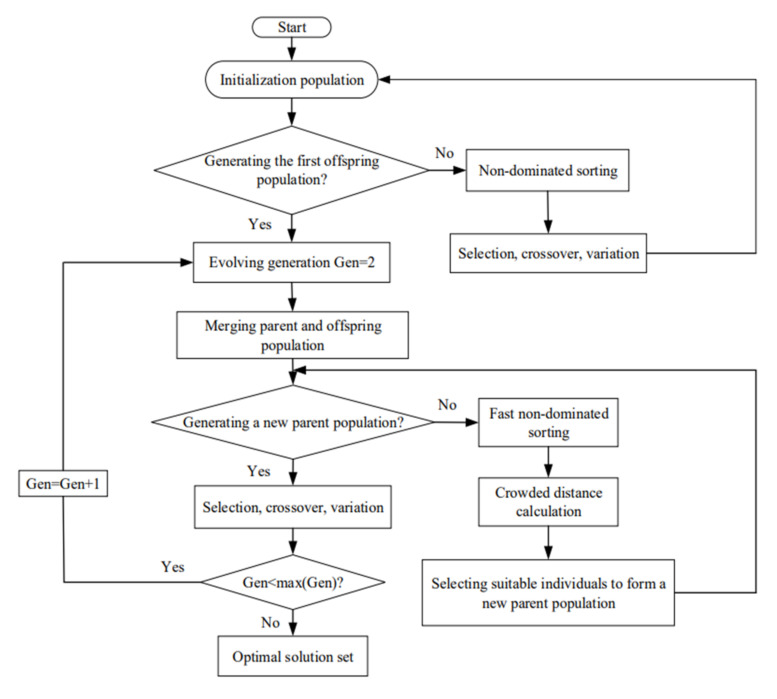
Flow diagram of NSGA-II.

With the increase in cycle OOs, the optimization of the cycle sometimes needs to take into account MOO. However, MOO cannot make many OOs achieve the highest value simultaneously. The finest compromise can be obtained by weighing the advantages and disadvantages of MOO. The NSGA-II (Figure 8 is the flow chart of the arithmetic) is applied herein, γ is taken as the optimization variables, and the ${\bar{P}}_{d}$, $\bar{P}$, η and $\bar{E}$ are taken as OOs, and one-, two-, three- and four-objective optimizations are performed. Three decision-making methods, LINMAP [90], TOPSIS [91,92] and Shannon Entropy [93], are used to select the reasonable solution, and the average distances (i.e., deviation index) [94] between Pareto frontier and positive or negative ideal point are compared, and the reasonable solution is obtained.

The deviation index is [94]

(32) $$D=\frac{\sqrt{\sum_{j=1}^{m}{\left(G_{j}-G_{j}^{\mathrm{positive}}\right)}^{2}}}{\sqrt{\sum_{j=1}^{m}{\left(G_{j}-G_{j}^{\mathrm{positive}}\right)}^{2}}+\sqrt{\sum_{j=1}^{m}{\left(G_{j}-G_{j}^{\mathrm{negative}}\right)}^{2}}}$$

where $G_{j}$ is the j-th optimization objective, $G_{j}^{\mathrm{positive}}$ is the j-th optimization objective of the positive ideal point and $G_{j}^{\mathrm{negative}}$ is the j-th optimization objective of the negative ideal point.

**Figure 9:**
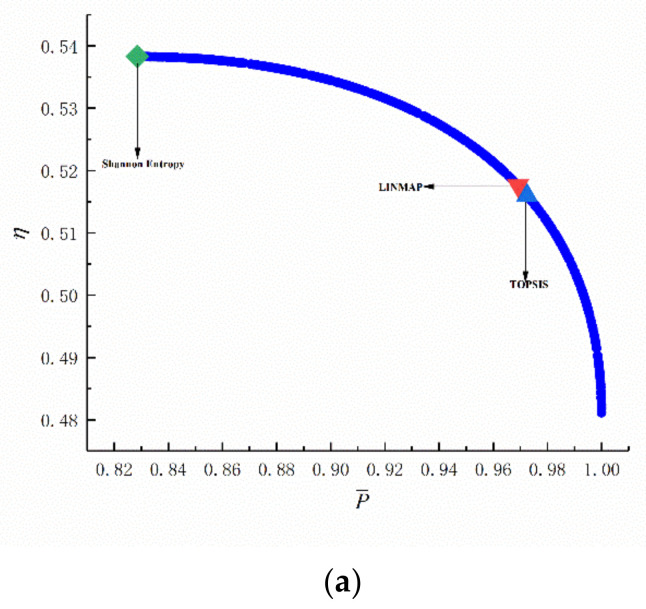

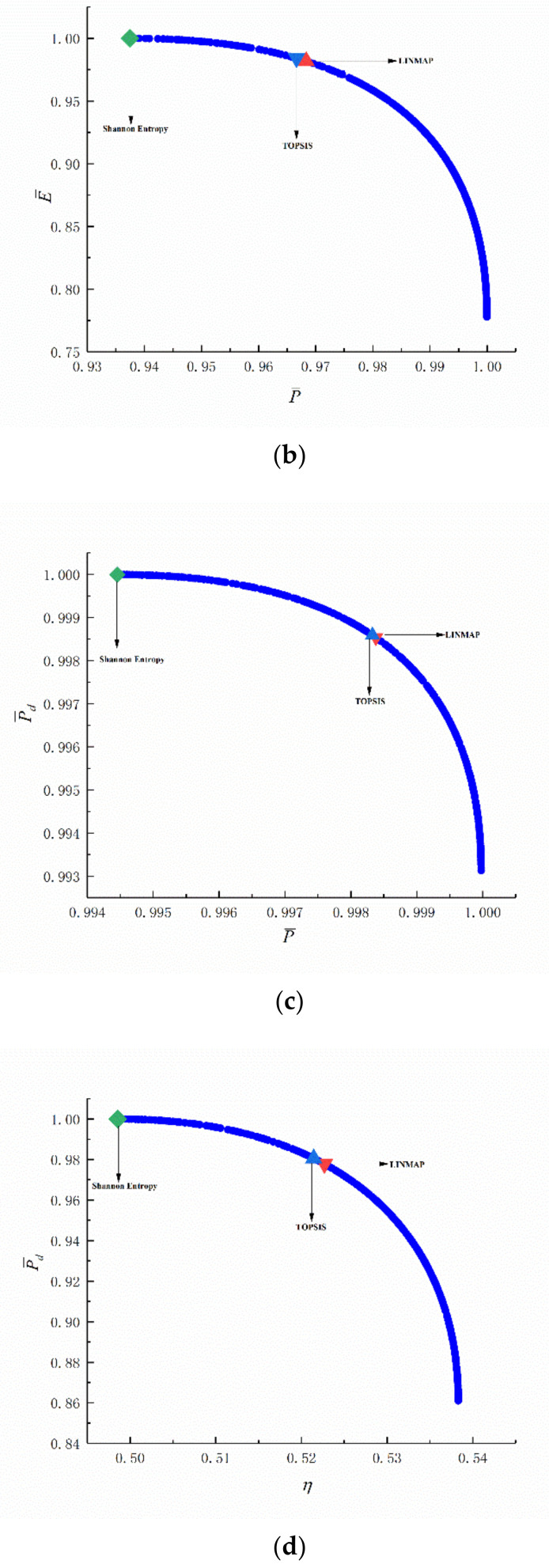

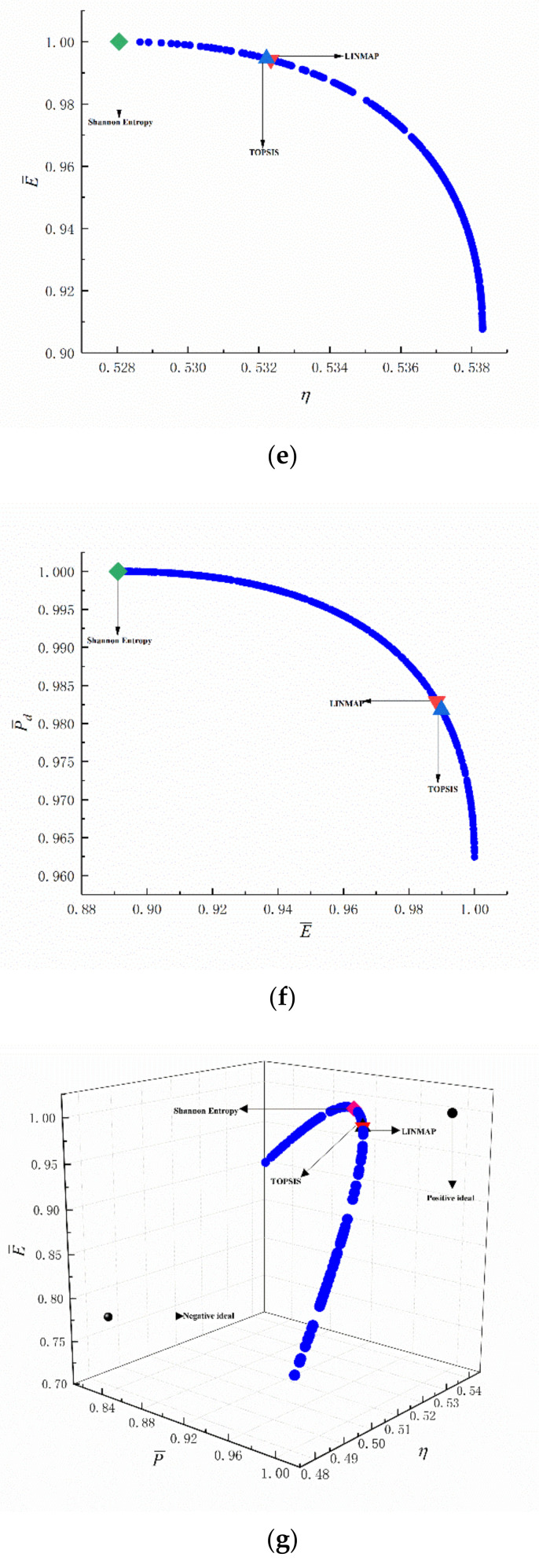

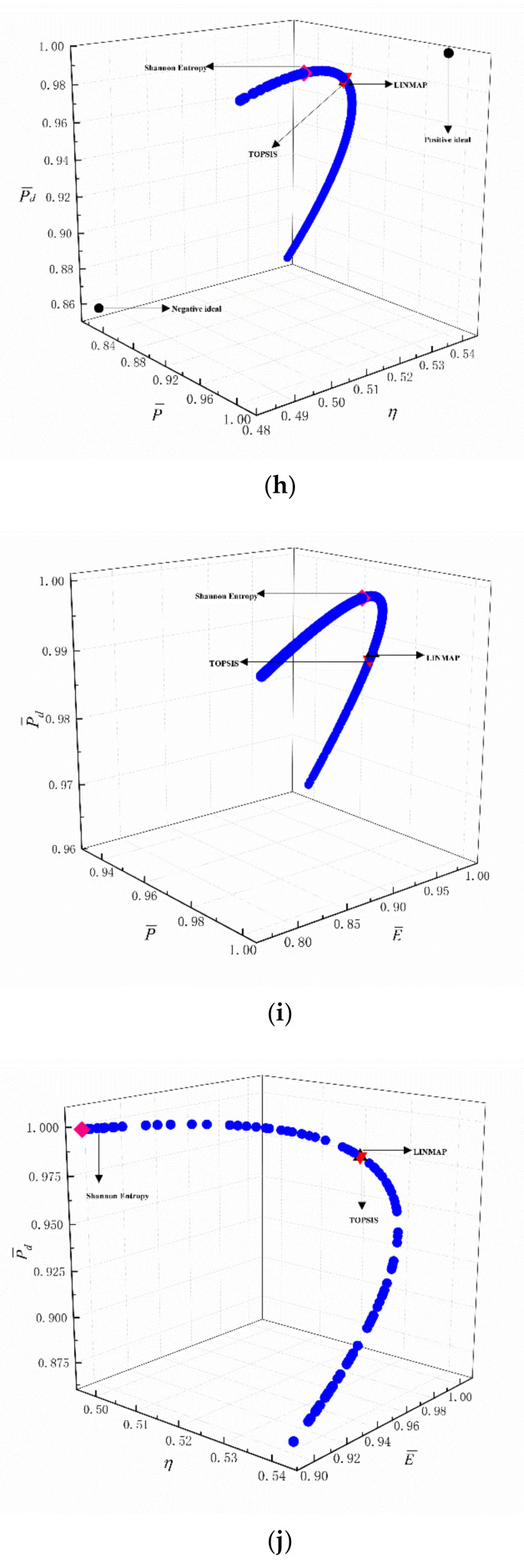

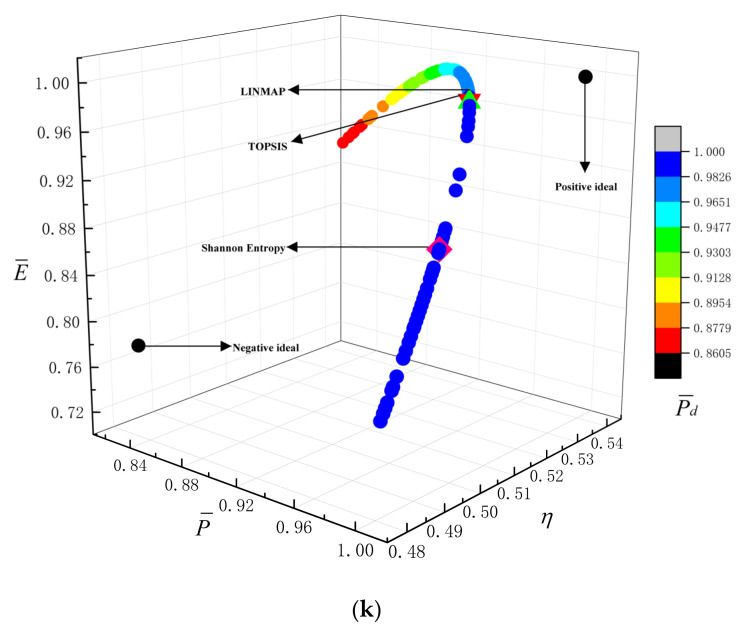
Multi-objective optimization results. (**a**) Two-objective optimization on $\bar{P}-\eta$. (**b**) Two-objective optimization on $\bar{P}-\bar{E}$. (**c**) Two-objective optimization on $\bar{P}-{\bar{P}}_{d}$. (**d**) Two-objective optimization on $\eta -{\bar{P}}_{d}$. (**e**) Two-objective optimization on $\eta -\bar{E}$. (**f**) Two-objective optimization on $\bar{E}-{\bar{P}}_{d}$. (**g**) Three-objective optimization on $\bar{P}-\eta -\bar{E}$. (**h**) Three-objective optimization on $\bar{P}-\eta -{\bar{P}}_{d}$. (**i**) Three-objective optimization on $\bar{P}-\bar{E}-{\bar{P}}_{d}$. (**j**) Three-objective optimization on $\eta -\bar{E}-{\bar{P}}_{d}$. (**k**) Four-objective optimization on $\bar{P}-\eta -\bar{E}-{\bar{P}}_{d}$.

**Figure 10:**
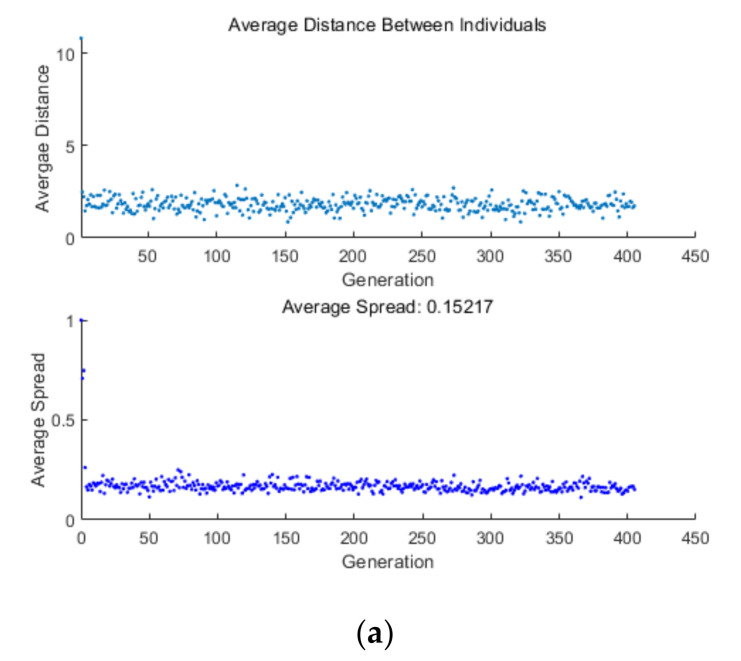

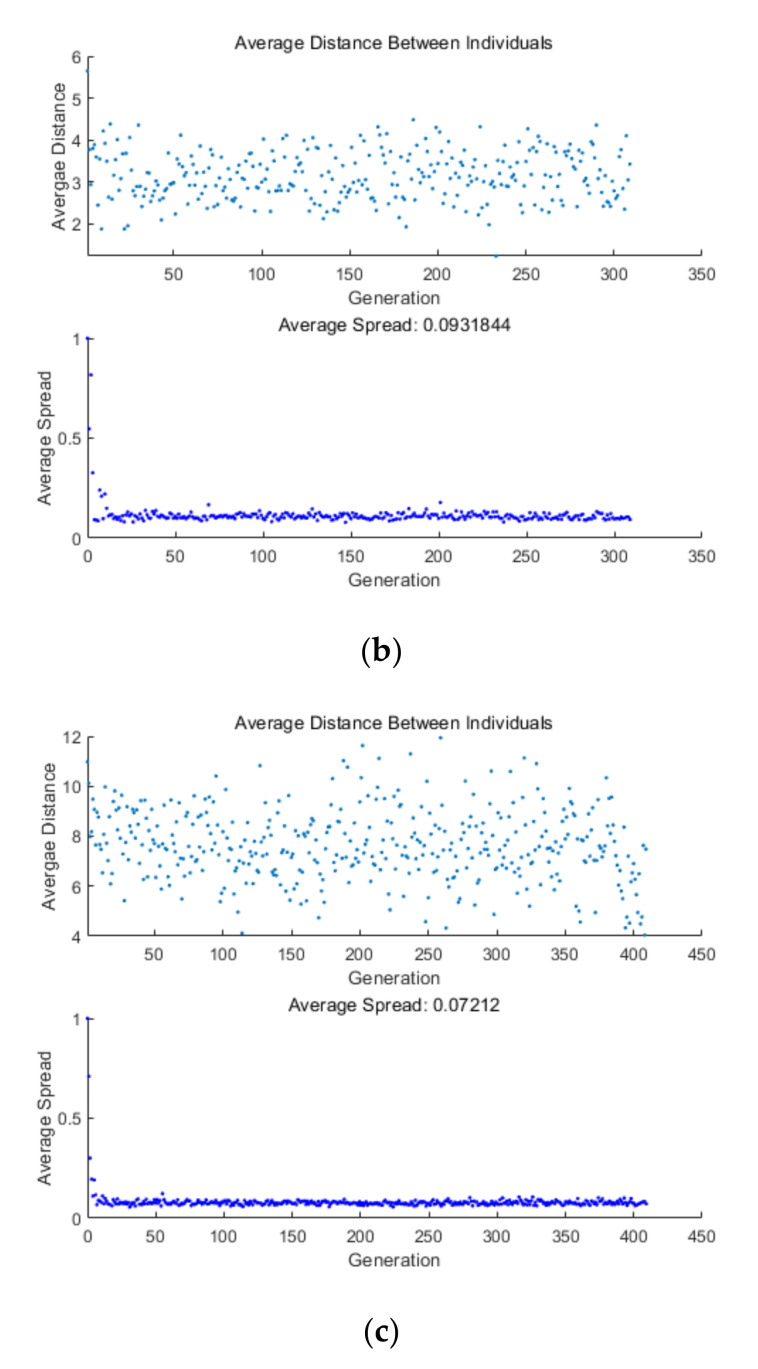
Average distance generation and average spread generation. (**a**) Average spread and generation number of $\bar{P}-\eta$. (**b**) Average spread and generation number of $\bar{P}-\bar{E}-{\bar{P}}_{d}$. (**c**) Average spread and generation number of $\bar{P}-\eta -\bar{E}-{\bar{P}}_{d}$.

Figure 9 shows the Pareto fronts for MOO, including six two-objective optimizations, four three-objective optimizations, and one four-objective optimization. Table 1 lists the numerical results. As seen in Figure 9a–f, as $\bar{P}$ grows, η, $\bar{E}$, and ${\bar{P}}_{d}$ decline. As η grows, ${\bar{P}}_{d}$ and $\bar{E}$ decline. As $\bar{E}$ grows, ${\bar{P}}_{d}$ declines. It can be seen from Table 1 that when $\bar{E}$ and ${\bar{P}}_{d}$ serve as the OOs, the DI obtained by the LINMAP is smaller. When $\bar{P}$ and η or $\bar{P}$ and $\bar{E}$ or η and ${\bar{P}}_{d}$ serve as the OOs, the DI obtained by the TOPSIS is smaller. When $\bar{P}$ and ${\bar{P}}_{d}$ or η and $\bar{E}$ serve as OOs, the DI obtained by the Shannon Entropy is smaller. In the two-objective optimization, when $\bar{P}$ and η serve as OOs, the DI obtained is the smallest. Figure 10a shows the average spread and generation number of $\bar{P}-\eta$ in the circumstances of two-objective optimization. The arithmetic converged at generation 395, and the DI is 0.128.

As seen in Figure 9g,h, as $\bar{P}$ grows, η declines, $\bar{E}$ and ${\bar{P}}_{d}$ first grow and then decline. As seen in Figure 9i, as $\bar{P}$ grows, $\bar{E}$ declines, and ${\bar{P}}_{d}$ first grows and then declines. As seen in Figure 9j, as η grows, ${\bar{P}}_{d}$ declines, and $\bar{E}$ grows first and then declines. It can be seen from Table 1 that when $\bar{P}$, η and ${\bar{P}}_{d}$ serve as OOs, the DI obtained by LINMAP is smaller. When $\bar{P}$, $\bar{E}$ and ${\bar{P}}_{d}$ serve as OOs, the DI obtained by TOPSIS is smaller. When $\bar{P}$, η and $\bar{E}$ or η, $\bar{E}$ and ${\bar{P}}_{d}$ serve as OOs, the DI obtained by the LINMAP and TOPSIS are the same, and both are smaller than the DI obtained by the Shannon Entropy.

In the three-objective optimization, when $\bar{P}$, $\bar{E}$ and ${\bar{P}}_{d}$ serve OOs, the DI is the smallest. Figure 10b shows the average spread and generation number of $\bar{P}-\bar{E}-{\bar{P}}_{d}$ in the circumstances of three-objective optimization. The arithmetic converged at generation 344 and the DI is 0.1353.

As seen in Figure 9k, as $\bar{P}$ grows, η declines, ${\bar{P}}_{d}$ grows, and $\bar{E}$ grows first and then declines. The DI obtained by the LINMAP is smaller. Figure 10c shows the average spread and generation number of $\bar{P}-\eta -\bar{E}-{\bar{P}}_{d}$ in the circumstances of four-objective optimization. The arithmetic converged at generation 304, and the DI is 0.1367.

It can be seen from Table 1 that when single-objective optimizations are carried out in the circumstances of $P_{\max}$, $\eta_{\max}$, ${\bar{E}}_{\max}$ and ${\left({\bar{P}}_{d}\right)}_{\max}$, respectively, the DI are 0.5448, 0.2897, 0.1960 and 0.2108, respectively, which are all larger than the best DI 0.1419 obtained in the four-objective optimization, which indicates that MOO produces better results.

## 5. Conclusions

Considering the linear variable SH characteristics of the WF, the optimal performance of irreversible PM cycle is studied with $P_{d}$ and E as the OOs in this paper. The effects of the parameters of the cycle on the $P_{d}$ and the E are analyzed; the corresponding η, $v_{1}/v_{s}$ and $p_{3}/p_{1}$ of the cycle under the conditions of ${\left(P_{d}\right)}_{\max}$ and $P_{\max}$ are compared; and the corresponding P and η of the cycle under the conditions of $P_{\max}$, $\eta_{\max}$, ${\left({\bar{P}}_{d}\right)}_{\max}$, and $E_{\max}$ are compared. The four OOs of the irreversible PM cycle are optimized with one-, two-, three- and four-objectives, respectively. The results show that:

1. The ${\bar{P}}_{d}-\gamma$ and ${\bar{P}}_{d}-\eta$ curves of the cycle are parabolic-like and loop-shaped, respectively. As the temperature ratio and pre-expansion ratio increase, three losses decrease and the specific heat of the working fluid changes more violently with temperature, the compression ratio and thermal efficiency in the circumstances of maximum dimensionless power density increase.
2. The $\bar{E}-\gamma$ and E-P curves of the cycle are parabolic-like and the E-η curves of the cycle are loop-shaped. As the temperature ratio and pre-expansion ratio increase, the compression ratio and thermal efficiency in the circumstances of maximum dimensionless ecological function increase. As three losses decrease and the specific heat of the working fluid changes more violently with temperature, the ecological function, power output and thermal efficiency increase.
3. Compared with the ${\bar{P}}_{\max}$ condition, the cycle in the circumstances of ${\left({\bar{P}}_{d}\right)}_{\max}$ is smaller and more efficient.
4. The DI obtained in one-objective optimization is larger than the optimal DI obtained in MOO, indicating that the MOO results are better. Comparing the results obtained by one-, two-, three- and four-objective optimization, the MOO corresponding to the double-objective optimization $\bar{P}-\eta$ is the smallest, and its design scheme is the most ideal.
5. Variable SH characteristics of the WF always exist. It is necessary to study its effects on the MOO performances of irreversible PM cycles.

## Figures and Tables

**Table 1:** Results of one-, two-, three- and four-objective optimizations.

Optimization Schemes	Solutions	Optimization Variable	Optimization Objectives				Deviation Index
		*γ*	$\bar{P}$	*η*	$\bar{E}$	${\bar{P}}_{d}$	*D*
Four-objective optimization ($\bar{P},\eta ,\bar{E}\mathrm{and}{\bar{P}}_{d}$)	LINMAP	25.9430	0.9664	0.5188	0.9844	0.9855	0.1367
	TOPSIS	26.2119	0.9650	0.5194	0.9861	0.9845	0.1380
	Shannon Entropy	19.2876	0.9944	0.4896	0.8914	1.0000	0.3216
Three-objective optimization ($\bar{P},\;\eta \;\mathrm{and}\;\bar{E}$)	LINMAP	26.9262	0.9612	0.5209	0.9902	0.9816	0.1443
	TOPSIS	26.9262	0.9612	0.5209	0.9902	0.9816	0.1443
	Shannon Entropy	31.1234	0.9374	0.5281	1.0000	0.9623	0.2137
Three-objective optimization ( $\bar{P},\;\eta \;\mathrm{and}\;{\bar{P}}_{d}$)	LINMAP	24.9370	0.9715	0.5165	0.9769	0.9891	0.1365
	TOPSIS	24.0989	0.9756	0.5144	0.9691	0.9918	0.1448
	Shannon Entropy	19.2843	0.9944	0.4986	0.8913	1.0000	0.3212
Three-objective optimization ($\bar{P},\;\bar{E}\;\mathrm{and}\;{\bar{P}}_{d}$)	LINMAP	25.1910	0.9703	0.5171	0.9789	0.9882	0.1355
	TOPSIS	25.4641	0.9689	0.5177	0.9810	0.9872	0.1353
	Shannon Entropy	19.2680	0.9945	0.4985	0.8909	1.0000	0.3220
Three-objective optimization ($\eta ,\;\bar{E}\;\mathrm{and}\;{\bar{P}}_{d}$)	LINMAP	28.1169	0.9547	0.5232	0.9952	0.9766	0.1602
	TOPSIS	28.1169	0.9547	0.5232	0.9952	0.9766	0.1602
	Shannon Entropy	19.2876	1.0000	0.4986	0.8914	1.0000	0.3173
Two-objective optimization ($\bar{P}\;\mathrm{and}\;\eta$)	LINMAP	25.3246	0.9696	0.5174	0.9800	0.9877	0.1353
	TOPSIS	27.7548	0.9724	0.5160	0.9939	0.9781	0.1281
	Shannon Entropy	25.5246	0.8285	0.5383	0.9815	0.9870	0.4126
Two-objective optimization ($\bar{P}\;\mathrm{and}\;\bar{E}$)	LINMAP	25.5543	0.9684	0.5179	0.9817	0.9869	0.1379
	TOPSIS	25.8498	0.9669	0.5186	0.9838	0.9858	0.1361
	Shannon Entropy	31.0929	0.9376	0.5280	1.0000	0.9625	0.2131
Two-objective optimization ($\bar{P}\;\mathrm{and}\;{\bar{P}}_{d}$)	LINMAP	17.5388	0.9984	0.4908	0.8437	0.9985	0.4170
	TOPSIS	17.5606	0.9984	0.4909	0.8444	0.9986	0.4157
	Shannon Entropy	19.2810	0.9944	0.4986	0.8912	1.0000	0.2934
Two-objective optimization ($\eta \;\mathrm{and}\;\bar{E}$)	LINMAP	34.8168	0.9151	0.5324	0.9941	0.9427	0.2896
	TOPSIS	34.5448	0.9168	0.5321	0.9949	0.9949	0.2336
	Shannon Entropy	31.1076	0.9375	0.5281	1.0000	0.9624	0.2134
Two-objective optimization ($\eta \;\mathrm{and}\;{\bar{P}}_{d}$)	LINMAP	27.7515	0.9567	0.5225	0.9938	0.9782	0.1549
	TOPSIS	27.1475	0.9600	0.5214	0.9912	0.9807	0.1469
	Shannon Entropy	19.2652	0.9945	0.4985	0.8909	1.0000	0.3220
Two-objective optimization ( $\bar{E}\;\mathrm{and}\;{\bar{P}}_{d}$)	LINMAP	26.6256	0.9628	0.5203	0.9886	0.9828	0.1413
	TOPSIS	26.8632	0.9616	0.5208	0.9898	0.9819	0.1435
	Shannon Entropy	19.2744	0.9945	0.4985	0.8911	1.0000	0.3216
$\mathrm{Maximum}\;\mathrm{of}\;\bar{P}$	——	15.7438	1.0000	0.4813	0.7788	0.9932	0.5135
Maximum of η	——	48.1678	0.8310	0.5383	0.9106	0.8631	0.6195
$\mathrm{Maximum}\;\mathrm{of}\;\bar{E}$	——	31.1146	0.9375	0.5280	1.0000	0.9624	0.2134
$\mathrm{Maximum}\;\mathrm{of}\;{\bar{P}}_{d}$	——	19.3173	0.9943	0.4987	0.8921	1.0000	0.3194
Positive ideal point		——	1.0000	0.5383	1.0000	1.0000	——
Negative ideal point		——	0.8287	0.4812	0.8000	0.8608	——

## Nomenclature

B	Heat transfer loss coefficient (W/K)
$C_{v}$	Specific heat at constant volume (J/(mol·K))
E	Ecological function (W/K)
k	Adiabatic index (-)
$\dot{m}$	Molar flow rate (mol/s)
P	Power output (W)
$P_{d}$	Power density (W/m^3^)
$\dot{Q}$	Heat transfer rate (W)
R	Gas constant (J/mol/K)
T	Temperature (K)
**Greek symbols**	
γ	Compression ratio (-)
η	Thermal efficiency (-)
$\eta_{c}$	Compression efficiency (-)
$\eta_{e}$	Expansion efficiency (-)
μ	Friction loss coefficient (kg/s)
σ	Entropy generation rate (W/K)
ρ	Pre-expansion ratio (-)
τ	Temperature ratio (-)
**Subscripts**	
in	Input
leak	Heat leak
out	Output
max	Maximum value
P	Max power output condition
η	Max thermal efficiency condition
$P_{d}$	Max power density condition
E	Max ecological function
1−5	State points
**Superscripts**	
−	Dimensionless
**Abbreviations**	
DI	Deviation index
FL	Friction loss
FTT	Finite time thermodynamics
HTL	Heat transfer loss
IIL	Internal irreversibility loss
MOO	Multi-objective optimization
OO	Optimization objective
PM	Porous medium
SH	Specific heats
WF	Working fluid
